# Mass spectrometry-based proteomics techniques and their application in ovarian cancer research

**DOI:** 10.1186/s13048-018-0460-6

**Published:** 2018-10-01

**Authors:** Agata Swiatly, Szymon Plewa, Jan Matysiak, Zenon J. Kokot

**Affiliations:** Department of Inorganic and Analytical Chemistry, Poznan University of Medical Sciences, Grunwaldzka 6 Street, 60-780 Poznań, Poland

**Keywords:** Ovarian cancer, Proteomics, Biomarkers, Drug-resistance, Diagnostics

## Abstract

Ovarian cancer has emerged as one of the leading cause of gynecological malignancies. So far, the measurement of CA125 and HE4 concentrations in blood and transvaginal ultrasound examination are essential ovarian cancer diagnostic methods. However, their sensitivity and specificity are still not sufficient to detect disease at the early stage. Moreover, applied treatment may appear to be ineffective due to drug-resistance. Because of a high mortality rate of ovarian cancer, there is a pressing need to develop innovative strategies leading to a full understanding of complicated molecular pathways related to cancerogenesis. Recent studies have shown the great potential of clinical proteomics in the characterization of many diseases, including ovarian cancer. Therefore, in this review, we summarized achievements of proteomics in ovarian cancer management. Since the development of mass spectrometry has caused a breakthrough in systems biology, we decided to focus on studies based on this technique. According to PubMed engine, in the years 2008–2010 the number of studies concerning OC proteomics was increasing, and since 2010 it has reached a plateau. Proteomics as a rapidly evolving branch of science may be essential in novel biomarkers discovery, therapy decisions, progression predication, monitoring of drug response or resistance. Despite the fact that proteomics has many to offer, we also discussed some limitations occur in ovarian cancer studies. Main difficulties concern both complexity and heterogeneity of ovarian cancer and drawbacks of the mass spectrometry strategies. This review summarizes challenges, capabilities, and promises of the mass spectrometry-based proteomics techniques in ovarian cancer management.

## Background

### Ovarian cancer

Ovarian cancer (OC) causes about 125,000 deaths each year, which corresponds to over 4% of women cancer deaths worldwide [1, 2]. Only 5–10% of the OC cases are hereditary [3, 4]. OC tumors generally originate from other gynecological tissues than ovaries. Interestingly, tumors involves the ovary tissue secondarily [5]. However, despite several hypotheses of the OC origin, understanding its pathogenesis is still insufficient. Therefore, it has become a widely researched topic in the field of molecular sciences, which may influence modern medicine. Unfortunately, even though progress is made in prevention, development of novel tools for early diagnosis and improvement of pharmacological therapies, the survival rate for OC remains poor. Patients often experience some symptoms but these are ignored or overlap with other ailments. Premalignant phase is difficult to recognize. A lack of sufficient screening options results in late detection. Despite successful surgery and appropriate treatment based on intravenous or intraperitoneal platinum- and taxane-based chemotherapy, diagnosis at advanced stages lowers 5-year survival rate to 27% [6]. This is caused by at least two factors: disease extension and biological differences in widely disseminated tumors [7]. Currently, less than 40% of all diagnosed OC cases are cured. However, if the diagnosis was made at the first stage of the disease, treatment could be limited to a surgical intervention alone [8].

### Proteomics in cancer biomarker discovery

The improvement in –omics sciences, genomics, proteomics, metabolomics, has opened a new research chapter, which is expected to develop novel tools for early diagnosis, treatment monitoring or population screening [9, 10]. Fundamentally, cancerogenesis is associated with a genetic defect and epigenetic changes [11]. Many studies suggest that germline mutations in Breast Cancer Gene 1 (BRCA1) (17q21, chromosome 17: base pairs 43,044,294 to 43,125,482) and Breast Cancer Gene 2 (BRCA2) (13q12.3, chromosome 13: base pairs 32,315,479 to 32,399,671) are associated with a risk of breast and ovarian cancer [12]. Moreover, in epithelial OC some sporadic BRCA1 and BRCA2 mutations may occur, including BRCA1 hypermethylation [13]. Currently, it is thought that BRCA could be a useful prognostic marker only in combination with other biomarkers [14]. Since proteins are expressed by genes, and they are functional factors in phenotype characterization, the study of proteome profiles may yield information crucial for cancer research. Predictive markers could increase our understanding of molecular processes and pathological mechanisms, which is a dire need in modern medicine [15]. Sporadic molecular mutations that occur during abnormal cellular proliferation result in changes in protein secretion, modification or degradation. Therefore, in-depth proteomics analysis of various biosamples (e.g., serum, plasma, urine, tissues) obtained from cancer patients may facilitate the study of tumorigenesis, therapy monitoring, and development of novel targeted treatments. However, biomarker discovery might be challenging, bearing in mind that biomarker should improve currently used diagnostic methods, increase their sensitivity and specificity, provide optimal treatment, correspond to disease stage, and also be easily available in biofluids [16].

So far, proteomics methods have revealed thousands of potential cancer biomarkers. Most of the proteins suggested in the literature as clinically useful molecules are still awaiting proper validation. Hypothesis-testing is one of the most critical aspects of the cancer research. Another challenge in biomarker discovery is standardization and optimization of protocols. Some approaches are characterized by low precision and reproducibility, which is associated with poor study design [17]. Moreover, there are also biological challenges like sample variability or cancer heterogeneity. Nevertheless, a few biomarkers have been successfully implemented into clinical practice. To date, American Food and Drug Administration approved Cancer Antigen 125 (CA125) and human epididymis protein 4 (HE4) as circulating OC biomarkers for therapy monitoring and recurrence identification [18]. However, these tests have some inherent limitations, and their sensitivity and specificity should be increased, especially for patients with early stage of the tumor.It has been proved that combination of existing biomarkers with additional markers in one discriminatory model may improve their performance [19]. Therefore, it may be suggested that proteomics is a chance to develop novel tools that will significantly reduce the OC mortality rate.

### Mass spectrometry techniques in clinical proteomics

The use of contemporary mass spectrometry (MS) represented a significant breakthrough in proteomics analysis. Since fast-evolving MS-techniques have a great impact on biomedical science, this innovative technology was introduced into clinical research [20]. Recently, matrix-assisted laser desorption/ionization (MALDI), and surface-enhanced laser desorption/ionization (SELDI) connected with time-to-flight (TOF) detector as well as electrospray ionization (ESI) have been extensively used in clinical proteomics [21–23]. Linking MS techniques with liquid chromatography (LC) or capillary electrophoresis allows for obtaining high resolution spectral proteomic patterns from numerous sample types [24–27]. These methods have already been reported as accurate tools for discovering multi-component classifiers, which significantly discriminate cancer samples from control biofluids or tissues. A crucial step in every analytical experiment is the choice of an optimal approach. Bearing in mind complexity of biological samples, the proper methodology should be chosen with respect to both sample pretreatment and MS-based strategy [28]. Initially, MS was used in clinical proteomics only to identify proteins and peptides. Today, the introduction of technical advances allows also for quantitative investigations.

**Fig. 1:**
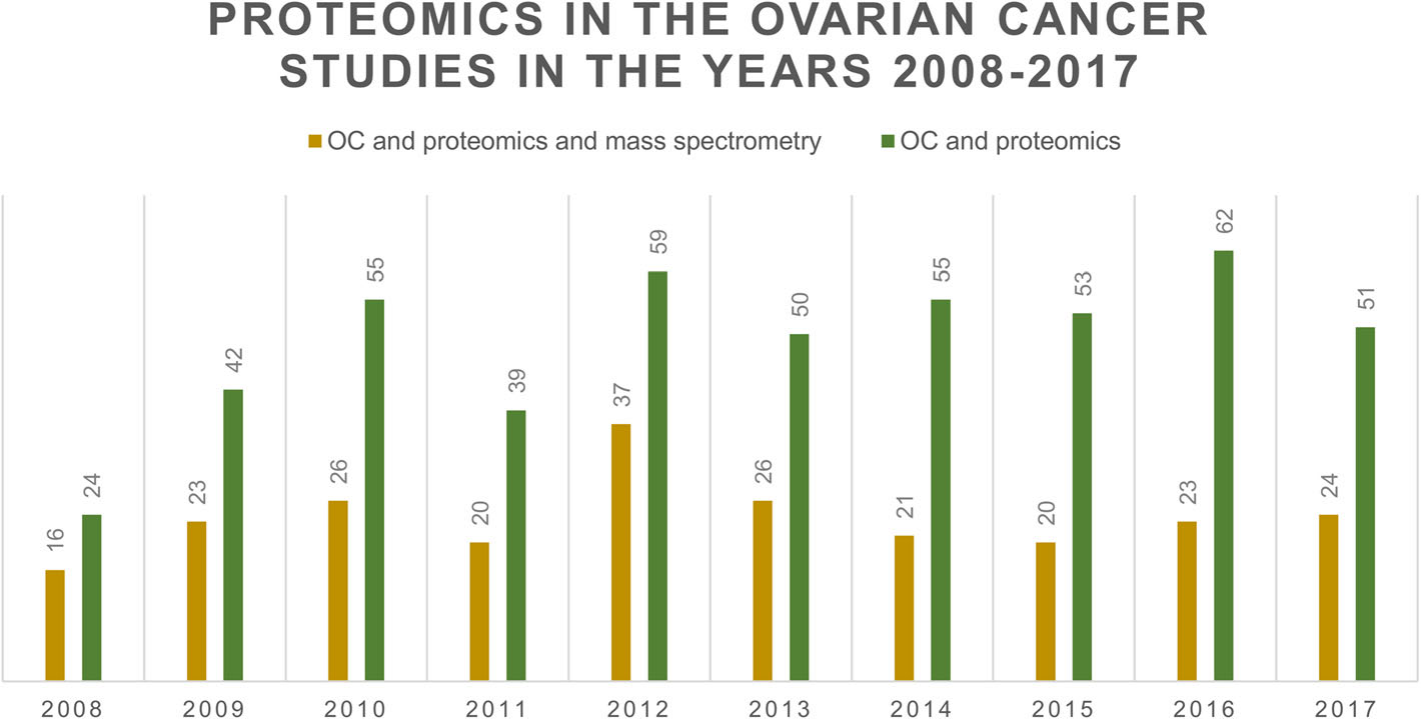
The use of proteomics and mass spectrometry in the ovarian cancer studies, according to PubMed

This review presents proteomics strategies based on MS techniques used in OC research. We summarize successes of the proteomics in OC management and discuss challenges associated with biomarker discovery and proteome analysis. Moreover, we discuss improvements in MS strategies and prospects for effective diagnosis and treatment of OC. The use of proteomics and mass spectrometry in the ovarian cancer studies in the years 2008–2017 is presented in the Fig. 1. It was prepared based on PubMed engine (http://www.ncbi.nlm.nih.gov/pubmed/) using the following keywords: “ovarian cancer and proteomics and mass spectrometry” and “ovarian cancer and proteomics”. In the years 2008–2010 the number of studies concerning OC proteomics was increasing, and since 2010 it has been approximately at the same level. While, the contribution of the MS techniques in these studies is significant over the years.

### Characterization of proteins in the OC development

The study of differentially expressed proteins in biosamples derived from OC patients or cell lines may improve early detection, treatment, and prognosis. MS-based proteomics techniques are widely employed to search sensitive and specific biomarkers, which, especially in combination with current diagnostic methods, may contribute to the detection of early stage OC. Moreover, identifying a relationship between overexpressed protein and dysfunctions in the cell cycle such as angiogenesis, apoptosis or proliferation, may be important for the development of novel therapies. Inhibition of the expression of significant proteins is a common method used to stop oncogenesis. Additionally, specific profiles of the proteins may indicate prognosis for the patients and help with proper treatment recommendations. Therefore, in this chapter, we discuss different MS-based strategies used in the clinical proteomics and their achievements in the field of the OC.

### Protein-peptide profiling

Petricoin et al. were the first who introduced low-molecular-weight protein profiling into cancer proteomics [29]. Discovery and identification of proteomic patterns that reflect specific health conditions have become promising tools in clinical investigations. Both MALDI-TOF and SELDI-TOF MS are soft ionization techniques that cause minimal fragmentation of the obtained ions. Therefore, they are mainly used for protein profiling in low mass range. The resulting profiles may contain even thousands of data points – registered ions (mass/charge ratio - m/z), which are subjected to sophisticated data analysis. Bioinformatic tools select the most discriminative m/z features, based on varying peaks intensities/areas, and define proteomic pattern characteristic for the study groups of samples. Subsequently, the differentiating peaks may be identified as proteins using tandem MS or protein databases. However, the identification process is omitted in some papers and the results are presented only as the m/z values [16]. Protein-peptide profiling offers various advantages, like a discovery of multi-marker panels, which usually demonstrate a higher level of discriminatory information comparing with single markers, or quick analysis of large groups of samples [30].

Protein-peptide profiling studies involve inexpensive and minimally invasive procedure of collecting blood and its components like serum or plasma. Moreover, other blood tests, which determine important factors and provide information about general patient condition, can be routinely performed in clinical laboratories. Biofluids represent the overall pathophysiological status of a studied individual [31]. Therefore, serum and plasma protein-peptide profiling has become a popular strategy in the field of ovarian cancer. In 2002, Petricoin et al. used SELDI-TOF MS to analyze serum samples from ovarian cancer patients and control group that contained healthy individuals and women with benign tumors [29]. A combination of a genetic algorithm and cluster analysis resulted in the selection of five m/z features: 534, 989, 2111, 2251, and 2465. The obtained discriminative panel significantly differentiated the study groups (sensitivity of 100%, specificity of 95%, and positive predictive value of 94%).

Further proteomic studies suggested that the use of a proper sample preparation method is required in order to detect aberrations in low abundance proteome. It is expected that potential biomarkers are mostly present at low concentrations, which disturbs their detection. For example, albumin, immunoglobulins, complement system proteins and transferrin are high-abundant blood proteins that represent about 99% of all serum proteins. Remaining 1% may be a rich source of yet unknown biomarkers [32]. To overcome the problem of high-abundance proteins, different strategies have been applied in OC biomarker discovery: immobilized metal affinity capture (IMAC) technology [33], immunodepletion [34], magnetic beads [35], solid-phase extraction [19], biomarker enrichment kits [30], and anion exchange chromatography [36]. Table 1 presents m/z ratios and proteins identified as potential OC serum biomarkers. Despite application of purification techniques, most of the identified proteins are still highly abundant ones, such as apolipoprotein A1 or complement component 3. Moreover, these proteins seem to be unspecific, as they are also differently expressed in various types of cancer [37, 38]. However, the features of these proteins might be used to create multivariate predictive models, especially in combination with two common OC biomarkers – CA125 and HE4. Recently, a new OC diagnostic test was proposed – OVA 1, which is based on determination of serum levels of five different factors: CA125, apolipoprotein A1, beta-2-macroglobulin, transferrin, and transthyretin [39]. Therefore, protein-peptide profiling studies may be not a proper strategy to discover single useful biomarkers but they could contribute to developing multifactor tests.

**Table 1:** M/z features (peptides) proposed as potential OC biomarkers in protein-peptide profiling studies

m/z	Identified protein	Ref.
1082.24; 1087.80; 1066.08; 1277.19; 1293.36; 1897.52; 4466.86; 4467.05; 4469.14; 4962.52; 8601.58; 8601.64; 11,693.29; 11,735.91; 17,105.23 5486; 6440; 13,720	no identification	[34] ᅟ ᅟ ᅟ [35]
28,043	apolipoprotein A1	[36]
12,828 2898.54 2210.80	transthyretin	[36] [30] [19]
3272 2582.35; 3027.57 2082.73; 3158.75	inter-α-trypsin inhibitor heavy chain H4	[36] [30] [19]
1041.68	keratin 2a	[30]
1224.68	glycosyltransferase-like 1B	[30]
1690.94; 1777.97; 1865.01; 2021.11 1505.24; 2023.17	complement component 3 precursor	[30] [19]
1739.93	complement component 4A preproprotein	[30]
1966.91	casein kinase II alpha 1 subunit isoform a	[30]
2115.05	D-amino-acidoxidase	[30]
2345.19	transgelin 2	[30]
3239.55	fibrinogen alpha chain isoform alpha preproprotein	[30]
1945.38	kininogen − 1	[19]

### Quantitative proteomics

Recently, new quantitative strategies of protein analysis have enjoyed huge attention. Since a discovery that proteomic changes under specific physiological conditions may help elucidate disease mechanisms and identify crucial biomarkers, a sensitive and accurate method for this purpose has been sought for. MS-based proteomics strategies may be divided into: “top-down” and “bottom-up” proteomics. In this review, we focus on the “bottom-up” strategy that is most common in clinical proteomics. The classical “bottom-up” strategy is based on staining proteins and two-dimensional electrophoresis. Stained spots of different abundance are digested, excised and identified mainly using MS techniques. Unfortunately, this strategy has many restrictions, as not all types of proteins are suitable for in-gel separation. Moreover, gel-to-gel variability made this method not significantly reproducible [40]. Due to these limitations, the classical approach seems to be often unreliable and quantitative analysis might be difficult to achieve. Therefore, the introduction of LC coupled with MS facilitated utilization of a novel “bottom-up” approach. A typical analysis starts with enzymatic (usually tryptic) digestion of the study proteins. Then the resulting short peptides are separated by LC, and eluates are further analyzed with tandem MS.

Recently two gel-free “bottom-up” strategies have been developed: isotopic labeling (like chemical isotopic labeling, isobaric tagging, metabolic isotopic labeling) and label-free analysis [41, 42]. In the label-based approach, peptides are linked with various tags in which ion signals correspond to relative peptide abundance in the analyzed sample. The most common labeling strategies include: SILAC (stable isotope labeling by amino acids in cell culture), ICAT (Isotope-coded affinity tag), iTRAQ (isobaric Tags for Relative and Absolute Quantification) and TMT (Tandem Mass Tags). Contrary to that, label-free analysis is based on precursor or spectral count. This approach has become popular due to a simpler sample preparation method and higher dynamic range as compared with labeling techniques [24]. The improvement of label-free approach consists in the advance of high-resolution mass spectrometer such as Orbitrap as well as TOF instruments [42].

**Fig. 2:**
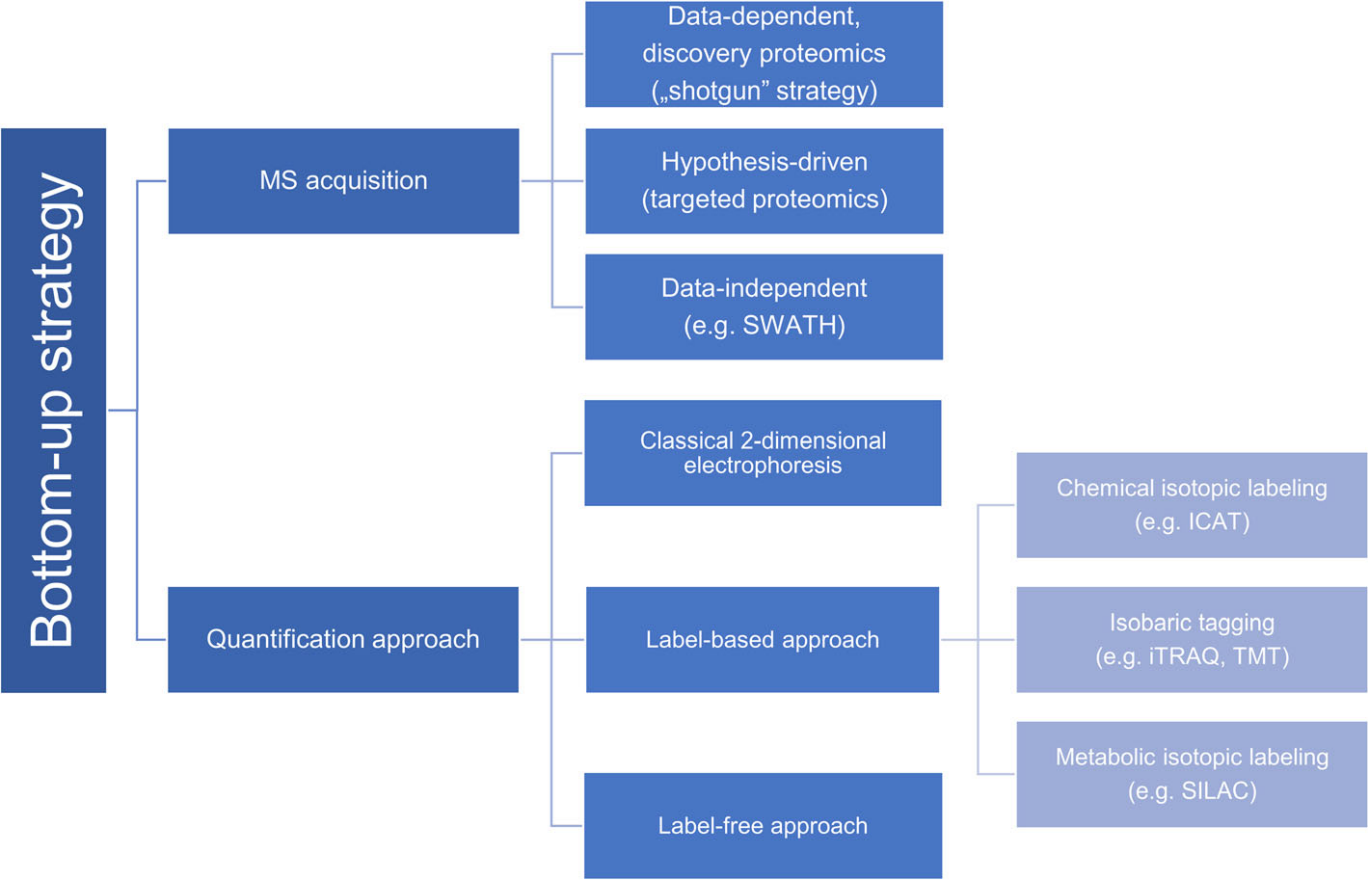
Division of “bottom up” strategy according to quantification approach and MS acquisition

Apart from the division into label-based and label-free methods presented in Fig. 2, two groups of “bottom-up” proteomics can be distinguished: discovery proteomics and targeted proteomics. The discovery proteomics is also called “shotgun” strategy, and it represents data-dependent acquisition. A considerable advantage of this method is a possibility to analyze thousands of proteins in one run. However, “shotgun” methods are usually characterized by low repeatability of peptide identification, which may be overcome by applying the targeted approach. Targeted proteomics techniques are mainly based on multiple-, selected- or parallel reaction monitoring, which results in sensitive and reproducible quantification of predefined proteins [42]. In the last few years, a third kind of acquisition has been developed: data-independent acquisition (DIA). During DIA analysis precursor ions from determined m/z isolation window are deterministically fragmented. Recently, sequential window acquisition of all theoretical mass spectra (SWATH) (exemplified by DIA) has become a popular approach for biomarker discovery based on spectral libraries and targeted data analysis [43].

Many different quantitative strategies have been applied in the ovarian cancer proteomics. The most commonly performed analysis of ovarian cell lines are based on SILAC strategy. Isotope label is bound directly to the proteins during their synthesis. One of the first studies in this field compared the usefulness of classical two-dimensional electrophoresis coupled with SILAC strategy for describing urokinase plasminogen activator influence on OC cells. It proved that labeling technique is characterized by low quantitative variation. Moreover, this research demonstrated that urokinase plasminogen activator is capable of changing the expression of some proteins like thioredoxin, annexin IV, and fatty acid binding protein 3 in the OC cells [44]. Another study suggested that calcium-activated chloride channel regulator 1 and chloride channels may be potential OC therapeutic targets [45]. SILAC strategy was also used to investigate oxidative stress in the OC cells, which is an important issue in developing novel photodynamic therapy agents [46]. It was also revealed that epithelial-mesenchymal transition activated by epidermal growth factor modifies metabolic processes and cell cycle control. Therefore, it is associated with OC development and progression [47].

Another approach widely used in OC characterization is isobaric tagging: TMT and iTRAQ [48]. These techniques allow for simultaneous identification as well as relative quantification of proteins in multiple samples. Isobaric tagging patterns are mainly focused on an analysis of proteolytic digestion of proteins and peptides. ITRAQ labeling combined with tandem MS techniques enabled a selection of a few potential OC biomarkers: serum amyloid A-4 [49], astacin-like metalloendopeptidase [49], protein S100-A11, Keratin type II cytoskeletal 8, inorganic pyrophosphatase, isocitrate dehydrogenase [50], legumain [51], and protein Z [52]. Moreover, iTRAQ analysis was proved to be a useful method to track changes in proteins during a transition from benign to malignant tumor-like PI3K/Akt signaling pathway [53]. Another study aimed at comparing protein expression of ovarian cancer and endometrial high-grade serous carcinomas in both tissue samples and cell lines. Both tumors exhibited similar protein profiles [54]. TMT is much less popular in the field of OC proteome. Sinclair J. et al. combined this technique with two-dimensional electrophoresis, two-dimensional LC, and MS techniques: MALDI-TOF, electrospray quadrupole time-of-flight (Q-TOF) and Orbitrap to analyze proteome of two OC cell line models. A set of potential OC biomarkers was proposed for further examination. Additionally, the utility of the selected biomarker – human active and pro-matrix metalloprotease-10, was examined using immunoassays to determine the level of this protein in human serum [55].

Although label-based strategies still remain the gold standard of quantitative proteomics, label-free methods have recently become more widespread. Therefore, LC combined with tandem MS was used to analyze plasma, ovary, and oviduct tissue samples from healthy and OC in chickens. The results were further compared to PCR and western blot analysis of human cell culture. Ovostatin 2 level was elevated in all the chicken samples as well as in human OC mRNA and cell lines [56]. The label-free analysis was also used in order to discover changes in protein expression and molecular/biological pathways in serum and tissues derived from malignant OC compared with benign tumors. Apolipoprotein AI and serotransferrin levels were reduced in both serum and tissue from patients with malignant OC tumor. Moreover, analysis of the interactome, which comprise the whole set of molecular interactions in a cell, and the pathways suggested a potential role of Poly(rC)-binding protein 1 in OC pathogenesis [57].

Although quantitative proteomics studies have significantly contributed to the discovery of OC protein patterns and cellular signaling networks, the approach has some limitations. SILAC method is mainly dedicated for cell line studies, which prevents human samples analysis [41]. The use of isobaric tagging patterns may be restricted by isotopic contaminations or background interference. Moreover, a common problem is co-isolation and fragmentation of some contamination ions together with the targeted precursor ion. Other drawbacks of the label-based approaches include high cost of the reagents and laborious sample preparation [16]. The major disadvantage of label-free methods is their low reproducibility. Additionally, they require extensive data processing and advanced statistical analysis [58].

### Post-translational modifications

One of the challenges of the proteomic analysis is the proteome complexity, which results not only from high dynamic range of the biological samples but also from a wide variety of post-translational modifications e.g. glycosylation, ubiquitination, or phosphorylation. Many studies have proved that various types of cancers are associated with aberrations in protein modifications. Therefore, recent studies have focused on this field of proteomics to provide novel information on the disease development. In order to detect post-translational modifications and define their structures, sensitive MS methods are required like MALDI-TOF or LC-ESI (ESI - electrospray ionization) combined with dissociation techniques [59].

Protein glycosylation is undeniably a common and complex modification, responsible for several biological processes, like cell communication, signaling, adhesion, protein folding or solubility [6, 60]. There are two main categories of glycosylation depending on glycan attachments: N- and O- glycans. Analysis and characterization of glycans may be challenging due to glycan variety and structure complexity [61]. To overcome this limitation, it is necessary to use targeted isolation and enrichment methods as well as sensitive detection techniques. Several methods have been proposed as glycoprotein affinity enrichment techniques: lectin chromatography, hydrazide chemistry, hydrophilic interaction liquid chromatography, or capture via immobilized titanium dioxide and boronic acid. Glycomics based on MS analysis has been successfully used to characterize the proteome of OC patients [60, 62]. A comparison of glycosyltransferases involved in N-linked pathway in OC and normal ovarian tissues allowed for identification of OC-specific glycoproteins and glycosylation aberrations. Selected glycoproteins in patient sera were verified using immunoprecipitation and microarray. Results pointed out periostin and thrombospondin as potential OC markers with cancer-specific glycosylation [63]. Furthermore, N-linked sialylated glycoproteins were examined in OC and healthy controls sera. It was suggested that haptoglobin, PON1, and zinc-alpha-2-glycoprotein might have specific sialylation aberrations of the glycopeptides in the OC samples [64]. Glycomic strategies were also used to analyze proximal fluids derived from OC patients: ascites and malignant ovarian cyst fluids. Sialome (sialic acid that contains glycoproteins) of OC was identified, including a set of 13 sialoglycopeptides proposed as novel biomarkers [65]. The importance of the post-translational research was demonstrated in a study focused on CA125 N-glycan forms. Differentiations between CA125 glycoforms from OC patients and controls may improve sensitivity and specificity of this widely used biomarker [66]. Recently, a novel methodology, coupling HILIC-UPLC and microarray for affinity-based study of cancer-associated glycans in OC cell lysates, was proposed. A structure of monoclonal A4 antibody was revealed. Understanding the antibody cancer-specific binding to glycans may simplify its use as a diagnostic indicator [67].

The intracellular signaling networks are mainly based on reversible protein phosphorylation. It is a highly dynamic post-translational modification responsible for e.g. metabolism, apoptosis, homeostasis or proliferation [68]. Phosphoproteins, like glycoproteins, are usually present in biological samples at low concentrations. Consequently, a number of enrichment strategies have been proposed: immunoprecipitation, immobilized metal affinity chromatography, metal-oxide affinity chromatography, chemical modification, magnetic beads or hydroxyapatite chromatography [68, 69]. Recent studies have revealed the potential of phosphorylation profiling in oncogenesis characterization. Proteomic strategies have resulted in the discovery of targets of kinase inhibitors [70]. However, this approach is still out of favor according to OC studies. Research published by Francavilla et al. described phosphoproteomics as a promising strategy for understanding molecular determinants of OC. An analysis of epithelial cells collected from OC and healthy patients showed that cyclin-dependent kinase 7 (CDK7) controls cell proliferation. Therefore, inhibition of CDK7 may contribute to the development of efficient therapeutic strategies [71].

Although post-translational modification analysis seems to be an accurate and effective strategy for biomarker discovery and understanding cell signaling networking, there are some limitations that should be considered. Firstly, the modifications have to be subjected to chemical or enzymatic release. For example, ionization of native glycans is very poor [60]. Generally, phospho- and glyco-proteins are present in biosamples at low concentrations, so sufficient enrichment methods are required [72]. Moreover, reliable study results usually require derivatization. Sample preparation might be an issue mainly due to post-translational modification heterogeneity [73]. Finally, MS techniques have a limited dynamic range and minor modifications cannot be detected. Even though, when proper detection is achieved, accurate spectra interpretation and protein structure construction are challenging [72].

### Protein and peptide tissue imaging

Human biofluids are usually characterized by high dynamic range, which complicates biomarker discovery process. As tissue samples are poor in proteins but rich in specific molecules related to disease, direct analysis of cancer tumors seems to be a promising approach. Standard tissue analysis is mainly based on immune-histochemistry (IHC) and histology. Nevertheless, deep proteomic tumor analysis may facilitate resolving the issues associated with OC diagnosis. The lack of significant biomarkers is not the only problem. Histological analysis usually confirms the disease and allows for tumor stage classification as per FIGO system. Further patient treatment depends on proper stage determination. However, staging is not sufficient as a prognostic parameter, and proper grade classification is an additional useful factor. Grading is assigned using light microscopy, which is rather subjective and not significantly reproducible. Therefore, new analytical methods are required to improve grading system and discover effective biomarkers. There are two rapidly evolving strategies to analyze proteins and peptides in tumor tissue: classical strategy and tissue imaging [74]. Classical proteomics such as LC-MS may be used to generate a molecular fingerprint of disease [75, 76]. These methods allow for identification of hundreds to thousands of proteins and peptides. However, the spatial distribution of specific molecules is lost in this kind of analysis as samples need to be homogenized. Recently, a direct MS analysis of tissue sections has become possible due to development of MALDI imaging mass spectrometry (MALDI-IMS) [77]. When coupled with histological analysis, MALDI-IMS may provide information not only about tissue proteome but also about the distribution of lipids, metabolites or xenobiotics. The use of MALDI technology requires a selection of matrix solution that coats tissue samples either with spray coating or with automatic matrix spotter. Firstly, MALDI-IMS was used to analyze fresh frozen tissue sections but this kind of material is usually not available during standard diagnostic setting. Thus, strategies focused on formalin fixed and paraffin embedded (FFPE) materials have been successfully adapted.

A number of MALDI-IMS analyses have been conducted in the field of OC. A comparison of MALDI profiling, MALDI-IMS, and IHC analysis enabled identification of Reg-Alpha Fragment of the 11S proteasome activator complex as a potential OC biomarker. The protein was present in OC tumor and absent in benign tissues [78]. Moreover, hierarchical cluster analysis was applied to prove that MALDI-IMS is effective in differentiation of tissue regions [79]. MALDI-IMS was also proved to be a useful strategy in the OC interface zone analysis. Plastin 2 and peroxiredoxin 1 were identified as upregulated proteins in tumor region as compared with a normal tissue [80]. Recently, a novel methodology focused on N-glycan analysis in FFPE OC tissue has been proposed. Imaging technology combined with ESI MS was used to generate images of N-glycan structure distribution in tissue sections [81]. Another interesting approach is a combination of MALDI-IMS with top-down microproteomics. An analysis of benign, tumor and necrotic-fibrotic regions derived from OC biopsy specimens allowed for detection of promising novel biomarkers. The proposed methodology may contribute to identification of proteins that are usually lost during conventional proteomics analysis [82].

MALDI-IMS is potentially a revolutionary technique in pathology. Recently, tremendous improvement has been made in the field of sample preparation and instrumentation. However, the technique still poses a few challenges. First, there are no studies confirming its utility in clinical settings. Second, sample preparation and measurement methodology should be proved to be reproducible and robust in different laboratories. For better understanding of MALDI-IMS results, the integration with other –omics studies like transcriptomics still needs improvement [77, 79].

### Proteomics in treatment response

Cytoreductive surgery coupled with chemotherapy based on paclitaxel, carboplatin, cisplatin, often in combination with taxanes, is the most common OC treatment option. A decision on postoperative chemotherapy depends on the risk of the tumor recurrence, stage, and grade. OC subtypes respond differently to treatment and chemo-resistance may occur as a serious side effect [6, 83]. According to statistics, even 25% of patients experience platinum resistance, and in 50–60% of cases cancer resistance develops during the treatment [84]. Investigation of chemotherapy mechanisms and discovery of factors responsible for chemotherapy response and resistance provide a selection of alternative therapies or chemo-sensitizing agents [85]. Unfortunately, individual agents offer rather low response rate: 5–20%. As a result, patients often have to choose between continuation of chemotherapy or supportive care only [84]. Drug resistance may occur due to pharmacokinetic, tumor-specific aberrations and microenvironment of the tumor [86]. Discovery of chemo-resistant biomarkers may improve personalized medicine and therapy planning. Therefore, a number of previously discussed techniques: MALDI-TOF MS, LC-MS/MS, SILAC, iTRAQ, label-free quantitation, ICAT, were proposed to identify new biomarkers and investigate proteome changes during OC treatment [85]. Previously identified chemo-resistance biomarkers are presented in Table 2.

**Table 2:** Drug-resistance markers in OC identified by MS-based proteomic techniques

Chemoresistance markers	MS technique	Ref.
Annexin 3; Destrin; Cofilin1; Gluthathione-S transferase omega 1; Cytosolic NADP+ dependent isocitrate dehydrogenase	MALDI-TOF	[87]
ATP synthase subunit alpha; Peroxiredoxin 3; Prohibitin; Electron transfer flavoprotein subunit alpha; Aldehyde dehydrogenase X	MALDI-TOF	[88]
ERp57	MALDI-TOF ESI-Q-TOF	[89]
Tumor rejection antigen (gp96) 1; Triose phosphate isomerase; Palmitoyl-protein thioesterase1 precursor; ER-associated DNAJ	MALDI-TOF ESI-Q-TOF	[91]
Aldehyde dehydrogenase 1 family, member A1; Annexin A1; Heterogeneous Nuclear Ribonucleoproteins A2; Rho GDP dissociation inhibitor	MALDI-TOF LC-MS/MS	[90]
Activated leukocyte cell adhesion molecule; A kinase anchoring protein 12; Nestin	Orbitrap	[92]
Thioredoxin domain containing 17	Orbitrap	[93]
Mitochondrial topoisomerase I	Orbitrap	[94]
Cell recognition molecule CASPR3; S100 protein family members; Junction adhesion molecule Claudin 4; CDC42-binding protein kinase beta	ESI-MS/MS	[95]
P53 binding protein 1; Catenin delta 1 and plakoglobin; EGF-containing fibulin-like extracellular matrix protein 1; Voltage-dependent anion-selective channel protein 1	HPLC-ESI-MS/MS	[96]
Pyruvate kinase isozymes M1/M2; Heat Shock Protein Family D	ESI-Q-TOF	[97]

MALDI-TOF MS strategy was first used to identify chemo-resistant biomarkers in a comparative analysis of platinum-sensitive and platinum-resistant cell lines. Five differentially expressed proteins were identified and further validated [87]. Another study focused on the same cell culture analysis discovered five new mitochondrial proteins potentially involved in chemo-resistance mechanisms. It was suggested that mitochondrial defects may be associated with drug resistance [88]. MALDI-TOF MS combined with LC-MS/MS analysis demonstrated that proteins responsible for metabolism, stress response, and apoptosis, are differentially expressed in paclitaxel sensitive and resistant OC cell lines. Moreover, one of disulfide isomerases was found to play an essential role in chemo-resistance [89]. Lee et al. also used a combination of two techniques: MALDI-TOF MS and LC-MS/MS and identified two potential chemo-resistant biomarkers in OC cells [90]. As glycosylation is often connected with cancer development, analysis of glycoproteins was used to discover useful biomarkers. Abnormalities in the expression of four glycoproteins were found to be characteristic of chemo-resistance cases [91].

A study utilizing label-free LC-MS/MS strategy confirmed that mitochondrial proteomic changes are important in both platinum [88] and cisplatin resistance [92]. Therefore, abnormalities in the concentration of three proteins were found to be characteristic of cisplatin-resistant OC cell line [92]. Moreover, the label-free approach confirmed that elevated levels of TXNDC17 in both OC cell and tissues are associated with paclitaxel resistance. The study additionally demonstrated the relationship between TXNDC17, poor prognostic factors, and short survival rate [93].

Recently, isotopic labeling has also been widely used in the investigation of drug resistance. SILAC analysis proved that, as in the case of platinum [88] and cisplatin resistance [92], doxorubicin resistance is partially caused by changes in mitochondria [94]. ICAT technique was applied in the study of cisplatin resistance. Changes in protein expression in chemo-resistant cells were compared with mRNA expression levels [95]. Furthermore, another study based on ICAT-MS/MS analysis integrated with RNA analysis pointed out 16 protein changes in the chemo-resistant OC tissues. These results triggered a conclusion that chemotherapy response and resistance are determined by a set of proteins from following classes: extracellular-matrix, junction or cell adhesion proteins [96]. ITRAQ strategy turned out to be a useful approach in multidrug-resistance investigations [97]. Moreover, iTRAQ technique revealed that major histocompatibility complex class I peptide repertoire of OC cells is associated with the pathological condition of the cell and may become a new treatment target also in chemo-resistant cancers [98]. Advanced bioinformatics approach contributed to establishing prediction models based on iTRAQ analysis results. It was proved that proteomic profiles of OC may provide information on platinum drug responses [99].

The studies on the chemoresistance mechanism are very promising and may improve the treatment and prognosis of patients with OC. However, there is still a lot of work ahead [86]. Firstly, many analyses are conducted only on cell lines. An appropriate clinical material is limited and difficult to obtain [85]. Therefore, all encouraging results should be verified using OC tissue. Despite high-throughput technologies, there is still no significant biomarker to predict a treatment response [85]. Moreover, many cancers may develop resistance to different drugs and therapies [6].

### Challenges for biomarker research in clinical proteomics

Although clinical proteomics did contribute to understanding of complicated molecular pathways of several diseases including OC, only few information and findings are significant enough to be translated into clinical settings. A great effort was made to introduce new, sensitive biomarkers to clinical practice. However, most of them did not meet the validation requirements. Currently, there seem to be a great number of studies focused on searching for marker candidates. Over the past decades, clinical proteomics has encountered some obstacles and challenges, which should be overcome in the future. In this chapter, we discussed the general challenges associated with the MS-based analysis of biomarkers. Drawbacks characteristic for the specific methods are presented in the particular chapters.

The first issue to deal with at the beginning of the research is experimental design. The sample size is often inadequate to draw any meaningful conclusions and to obtain significant statistical power. Selection and building of suitable statistical methods and models is an essential part of the experiment. Moreover, in order to ensure the necessary impact of the analysis, the study groups should be properly selected. Factors such as age, diet, medication, etc. may have a crucial influence on the experiment [100]. Discovery of a sensitive and specific OC biomarker requires analysis and comparisons of several groups of samples including healthy controls, inflammatory controls, benign adnexal masses, early OC stage, and preferably other malignancies than OC. Proteins selected as relevant OC markers should be further compared with CA125 and HE4, and their utility as OC markers should be confirmed in longitudinal monitoring. Finally, for proper assessment of sensitivity and specificity, the selected markers need to be applied to blinded large study sets. Most of the experiments include only pilot analysis and omits another important step – expanding the study groups and sample size.

Another crucial factor in every proteomic experiment is the quality of clinical samples quality. The best way to collect samples is to create a biobank with all epidemiologic and genetic data. Samples should be collected in the conditions compatible with MS method and immediately frozen (e.g., in liquid nitrogen), preferably with the addition of protease inhibitors. One of the main pitfalls is the influence of inappropriate storage temperature and freeze-thaw cycles on the protein degradation. Endogenous contaminations with salts or lipids, and even inappropriate type of sample tube may affect the result of the experiment [101]. Therefore, in order to obtain the highest sample quality, a good communication between proteome scientists and clinical staff is necessary and all aspects of samples collection should be discussed.

Over the past years, unsuccessful efforts have been undertaken to discover OC screening method, including a unique proteomic signature. One of the main reasons for ineffective investigations is the limitations of MS methods. Despite significant progress in MS techniques, high dynamic proteome range still occurs as a major challenge. The most relevant proteins are usually present in biological samples in low concentrations, and their detection remains very difficult when high abundant proteins coexist. The most frequently used types of sample are serum and plasma. Since ten high abundant serum/plasma proteins account for about 90% of the total proteins, examination of all low concentrated proteins – the promising source of biomarkers is a huge challenge. To reduce the overall sample complexity, several methods have been proposed, including pre-fractionation, depletion or enrichment. However, proteomic techniques are still not sufficient to detect low abundant molecules. Although they are able to simplify the protein mixture, major blood components remain highly concentrated. Moreover, some depletion methods and other preanalytical steps lead to the removal of relevant proteins due to non-specific-bounds [16]. Therefore, the issue of low-abundance proteins analysis should be studied in the near future.

Even though MS-based techniques have been used in proteome science for a long time, there are still no uniform workflows and procedures. Therefore, inter-laboratory variability and low reproducibility often occur [16]. Another analytical challenge is statistical analysis and interpretation of the results containing hundreds, sometimes even thousands, of peptides and proteins. A typical result of the proteomic experiment is a list of interferences containing peptide-spectrum matches. Each interference is characterized by a score, which shows the confidence of correct identification. However, to avoid false-positive results, significant bioinformatics strategy may be a crucial step. *P*-value is not suitable for multiple testing of a database search, and need to be improved, usually by false discovery rate calculations [102]. Moreover, multiple comparisons require the use of proper corrections (e.g., Bonferroni correction, determination of the q-value) [72].

Past decade of MS-based research revealed many obstacles associated with biomarker discovery. Thus, more restrictions are required for each further analysis. Every experiment data should be validated with other complementary method like ELISA or western blot. Further analysis need to be also conducted using an external set of samples to ensure the reliability of the obtained results. Moreover, presented studies should be no longer limited to the list of differentially expressed proteins and peptides. Identified molecules need to be connected into one network and they need to explain complicated disease mechanisms. These steps are necessary for the future implementation of biomarker assessment to clinical practice.

Despite many challenges in biomarker studies, there are already two multimarker tests: OVA 1 and ROMA (risk of ovarian malignancy algorithm) approved by the Food and Drug Administration for OC diagnosis. Therefore, it should be expected that the current studies constitute a solid basis for creating new reliable diagnostic tools in the future. The addition of sophisticated MS techniques, like MALDI-IMS, to methods commonly used in the clinics, may be a step forward to significantly improve diagnosis and treatment.

## Conclusions

**Fig. 3:**
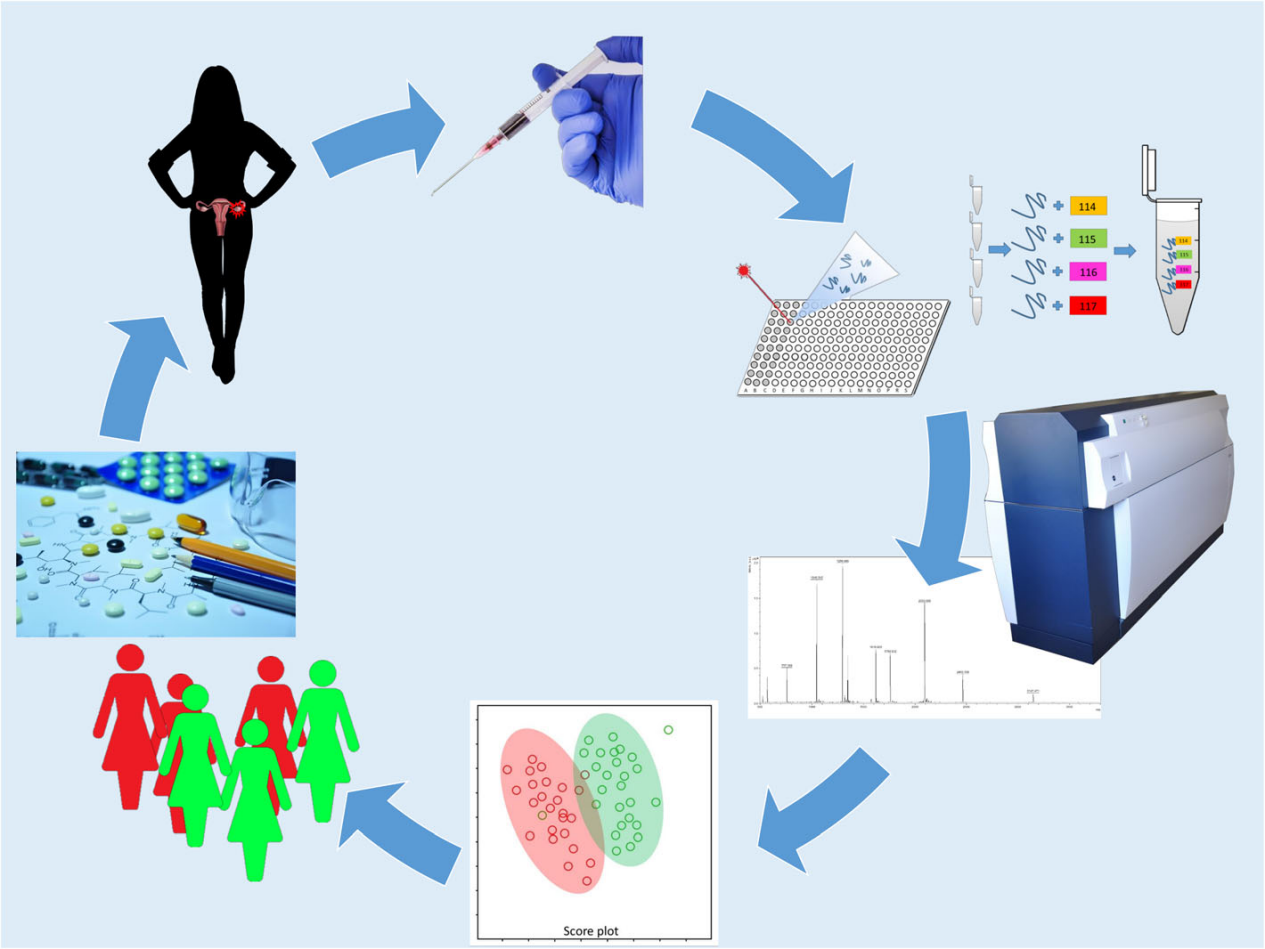
Development of novel, innovative diagnostics methods, therapies or drug response monitoring based on proteomics techniques

Since OC is responsible for thousands of deaths each year, there is a dire need to improve diagnostics as well as treatments strategies. Elucidating the mechanisms and molecular pathways during carcinogenesis may contribute to developing novel targeted therapies and personalized medicine. Proteomics, as a rapidly evolving branch of science, may be essential in novel biomarkers discovery, therapy decisions, progression prediction, and monitoring of drug response or resistance. This review presents main proteomics techniques based on MS that provide information on molecular mechanisms of the OC. Many limitations of particular approaches have also been discussed. The first challenge is the heterogeneity and diverse origins of the OC. It should be taken into account during basic and clinical studies design. Another difficulty is a limited access to biological samples, especially OC tissues. Studies based on the OC cell lines should be verified using tumor tissues. The next issue that should be considered is low inter-laboratory reproducibility of the results. There are many studies proposing different OC biomarkers but usually none further research is conducted to confirm the proposals. Moreover, there are still some improvements necessary in MS techniques. Analytical protocols need to be standardized to introduce a reproducible and high-throughput analysis. Lack of uniform workflow leads to false-positive results associated for example with sample collection errors. Moreover, high dynamic range of compounds in biological samples is an issue that is still not solved. A great challenge for proteome scientists is also developing significant bioinformatics tools and proper statistics strategies. Another important obstacle, frequently ignored, is validation of the obtained results and data interpretation that lead to understanding of complicated molecular pathways connected with the disease. However, current studies are getting more and more sophisticated. Due to multidisciplinary teams and close cooperation of clinical staff and scientists, the study design becomes more advanced. Authors believe that considering fast development of MS techniques, all drawbacks may be overcome in the next few years. Although the proteomics strategies still require optimization, they have also a lot to offer and in the near future they may provide insights into complicated and inaccessible cancer proteomics, which is presented in the Fig. 3.

## Abbreviations

BRCA 1: Breast Cancer Gene 1
BRCA 2: Breast Cancer Gene 2
CA125: Cancer Antigen 125
CDK7: cyclin-dependent kinase 7
DIA: data-independent acquisition
ESI: electrospray ionization
FFPE: formalin fixed and paraffin embedded
HE4: human epididymis protein 4
ICAT: Isotope-coded affinity tag
IHC: immune-histochemistry
IMAC: immobilized metal affinity capture
IMS: MALDI imaging mass spectrometry
iTRAQ: isobaric Tags for Relative and Absolute Quantification
LC: liquid chromatography
m/z: mass/charge ratio
MALDI: matrix assisted laser desorption/ionization
MS: mass spectrometry
OC: ovarian cancer
Q-TOF: quadrupole time-of-flight
ROMA: risk of ovarian malignancy algorithm
SELDI: surface-enhanced laser desorption/ionization
SILAC: stable isotope labeling by amino acids in cell culture
SWATH: sequential window acquisition of all theoretical mass spectra
TMT: Tandem Mass Tags
TOF: time-to-flight
